# Modeling the Chemoelectromechanical Behavior of Skeletal Muscle Using the Parallel Open-Source Software Library OpenCMISS

**DOI:** 10.1155/2013/517287

**Published:** 2013-11-17

**Authors:** Thomas Heidlauf, Oliver Röhrle

**Affiliations:** ^1^Universität Stuttgart, Institut für Mechanik (Bauwesen), Lehrstuhl II, Pfaffenwaldring 7, 70569 Stuttgart, Germany; ^2^Stuttgart Research Centre for Simulation Technology, Pfaffenwaldring 5a, 70569 Stuttgart, Germany

## Abstract

An extensible, flexible, multiscale, and multiphysics model for nonisometric skeletal muscle behavior is presented. The skeletal muscle chemoelectromechanical model is based on a bottom-up approach modeling the entire excitation-contraction pathway by strongly coupling a detailed biophysical model of a half-sarcomere to the propagation of action potentials along skeletal muscle fibers and linking cellular parameters to a transversely isotropic continuum-mechanical constitutive equation describing the overall mechanical behavior of skeletal muscle tissue. Since the multiscale model exhibits separable time scales, a special emphasis is placed on employing computationally efficient staggered solution schemes. Further, the implementation builds on the open-source software library OpenCMISS and uses state-of-the-art parallelization techniques taking advantage of the unique anatomical fiber architecture of skeletal muscles. OpenCMISS utilizes standardized data structures for geometrical aspects (FieldML) and cellular models (CellML). Both standards are designed to allow for a maximum flexibility, reproducibility, and extensibility. The results demonstrate the model's capability of simulating different aspects of nonisometric muscle contraction and efficiently simulating the chemoelectromechanical behavior in complex skeletal muscles such as the tibialis anterior muscle.

## 1. Introduction

Skeletal muscles' ability to actively generate force in a controlled fashion allows us to consciously move our body. The force generation is achieved through complex processes on multiple scales and multiple parts of the musculoskeletal system, for example, neural stimuli generation, depolarization at neuromuscular junctions, force generation within skeletal muscle sarcomeres, force transmission to the tendons, and sensory feedback to the nervous system. These processes are extremely complex, strongly coupled with each other, and by far not fully understood. Like in many other research areas, detailed simulation frameworks appealing to realistic models can provide an effective tool to investigate functional and structural interrelations of skeletal muscle force generation. An improved understanding of the physiological mechanisms may also lead to a better understanding of mechanisms behind musculoskeletal disorders.

State-of-the-art simulations taking into account the force generating capabilities of skeletal muscles are subject to either phenomenological descriptions using discrete [1–4] or continuum-mechanical models [5–7]. The most commonly used skeletal muscle modeling frameworks investigating the musculoskeletal system as a whole are based on discrete mechanics, that is, rigid-body dynamics simulations, in which the skeletal muscles are described by Hill-type models (cf. the review by Zajac [8]). Although such models are widely used to analyze movement, they exhibit significant drawbacks. All functional and structural properties are lumped together to a few parameters. For example, Hill-type skeletal muscle models are described at a point in space through spring constants, damper properties, and one overall activation level, and the calculated muscle force acts along a predefined line of action. Since such models lack a volumetrical representation of the skeletal muscles, they are not capable of properly taking into account structural properties, for example, complex fiber architectures, motor unit fiber distributions, or the interaction of a skeletal muscle with surrounding tissue, for example, bones, muscles, or fat tissue.

While continuum-mechanical skeletal muscle models can take into account complex muscle fiber distributions [9], regional activation properties, and a dynamically generated line of action [7], they are computationally more challenging and restrict their findings purely to mechanical aspects of muscle force generation; for example, see [6]. Further, researchers appealing to continuum-mechanical models mainly focus on skeletal muscles in isolation. However, considering natural motor unit (MU) recruitment principles to activate specific skeletal muscle fibers by action potentials (APs, electrical signals of short duration), one has to replace such single scale continuum-mechanical models with multiscale, multiphysics models that take into account the entire pathway from neural stimulation to muscle force generation and feedback to the neural system.

Models describing the excitation-contraction coupling (ECC) do exist [10, 11] but are typically limited to describe the force generation within a sarcomere and, hence, on the cellular level and not on the level of an entire skeletal muscle. Models that are guided by either the natural principles of MU recruitment, MU fiber distributions, or muscle force generation on the cellular level and its effect on the force generation of an entire skeletal muscle are rare and do often have significant limitations. For example, Hernández-Gascόn et al. [12] include a phenomenological description of the cellular processes and ignore biophysical principles of AP propagation and crossbridge dynamics. Fernandez et al. [13] use a neuron model to simultaneously generate an AP at all neuromuscular junctions that is propagated through the muscle tissue using the three-dimensional (3D) bidomain equations neglecting functional structures such as MU fiber distributions or the fact that APs propagate along a single muscle fiber and do not effect neighboring ones. Furthermore, the model describing the cellular behavior of a sarcomere was adopted from cardiac mechanics. Böl et al. [14] couple 3D electrical field equations with phenomenological fiber models. The model of Röhrle and coworkers is currently the only one that can take into account a biophysical cell model, which includes multiple subcellular models including fatigue, and allows for spatial descriptions, MU fiber distributions, MU recruitment principles, and skeletal muscle force generation [15–18].

However, the chemoelectromechanical model of Röhrle and coworkers has framework-inherent limitations that do not allow its extension to a fully coupled framework embracing neural inputs, force generation, and feedback mechanisms. The major limitation is the fact that the cellular equations are only unidirectionally coupled to the mechanical model. The behavior of a single skeletal muscle fiber is precomputed and stored in a look-up table. Within the mechanical model, the cellular variables associated with force generation, that is, the crossbridge concentrations in the attached pre- and postpower stroke state (*A*_1_ and *A*_2_, resp.), are copied into a detailed 3D structural model and homogenized to compute the resulting stress tensor. Any geometrical variations of a skeletal muscle fiber due to a contraction, for example, a length change, are not considered. The same applies to feedback, that is, an alternation of the recruitment sequence due to the mechanical state. The choice of precomputing the cellular behavior has been chosen to reduce the overall computational cost. This was necessary as the original framework is based on serial legacy code (CMISS) appealing to data structures not necessarily suitable for parallelization. Further, only isometric contractions were considered. The isometric case provided justification for neglecting the force-velocity relationship. In reality, however, series elastic elements against which the muscle shortens during tension development prevent true isometric conditions.

The aim of this contribution is to introduce a completely new, computationally efficient, fully coupled, multiphysics simulation framework for skeletal muscle modeling providing the basis to include biophysical motor unit recruitment and feedback mechanisms at a later stage. The framework is based on the open-source software library OpenCMISS [19], which, together with the entire model described in this contribution, can be downloaded from https://github.com/OpenCMISS. OpenCMISS was designed to achieve maximal flexibility and efficiency through the use of new data structures such as FieldML [20], access to well-established model repositories via CellML [21, 22], and a distributed-memory foundation for executing large problems. The new libraries and the data structure provide the basis to combine different mesh regions with different dimensionality, for example, 0D models for the cellular behavior, 1D models for the AP propagation, and 3D models for the mechanical model, within one framework. This allows for a strong and bidirectional coupling of the chemoelectrical cellular behavior and the mechanical model—a major advantage over commercially available software packages. Furthermore, the modular organization of the framework allows for straightforward extensions of the model and substitution of model components, for example, the cellular model.

## 2. Materials and Methods

**Figure 1:**
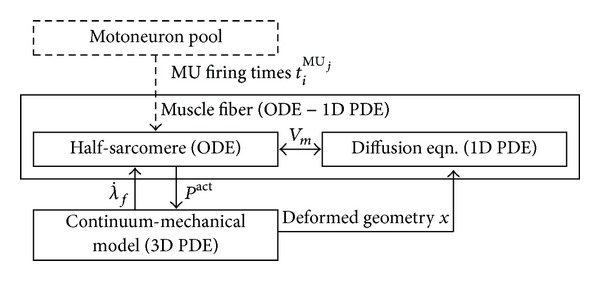
Overview of the modeling framework. Each box indicates a model part. The couplings between the parts are indicated through arrows together with the transferred information. The membrane voltage, *V*_*m*_, couples the half-sarcomere model to the diffusion equation. Together, they represent the electrophysiological behavior of the muscle fiber. Further, ${\dot{\lambda }}_{f}$ is the shortening velocity, and *P* ^act^ is the active stress. The model of the motoneuron pool is not part of the proposed model, which is symbolized by a dashed box.

Figure 1 provides an overview of the proposed computational framework. The individual parts of the framework (model of the half-sarcomere, propagation of the AP, and continuum-mechanical model) are presented in the subsequent sections. Here, the interactions and couplings between the individual model parts are explained. 

The muscle fibers of one MU are stimulated through their corresponding motoneuron at the neuromuscular junction. In the proposed model, the neural discharges are modeled as an ionic current that is applied at the center of a fiber, which represents the neuromuscular junction. In this contribution, the MU discharge times are predefined, for example, by a regular frequency. However, computing the discharge rates of a motoneuron pool, one could, for example, also appeal to the model of Fuglevand et al. [23], as shown in Röhrle [16], or to a biophysical model like the one by Negro and Farina [24]. The coupling of a motoneuron-pool model to the muscle model is unidirectional; that is, the flow of information between the models only occurs from the motoneuron-pool model to the muscle model. Hence, the MU recruitment and firing times can be precomputed independently of the muscle model.

In contrary to [15], the governing equations for describing the bioelectrical field and those of 3D finite elasticity theory are solved in a strongly coupled way, where the solution of the mechanics influences the bioelectrical fields and vice versa.

The bioelectrical field itself is determined by solving the so-called monodomain equation, which is a reaction-diffusion equation. The monodomain equation is solved using an operator splitting technique providing the mathematical justification to separately treat the reaction part, which is given by the half-sarcomere model [11], and the diffusion part, which describes the AP propagation. The half-sarcomere model is biophysically based and is described by a set of ordinary differential equations (ODEs) in time, that is, exhibiting no spatially varying quantities (0D). The diffusive part is described by a transient 1D partial differential equation (PDE). Since the two parts of the monodomain equation are solved separately, the operator splitting technique requires a mapping of the membrane voltage, *V*_*m*_, between the two parts at each time step. Among many other cellular quantities, the half-sarcomere model (reaction term) computes the active stress contribution at a particular location along a skeletal muscle fiber. The active stress, *P* ^act^, enters the constitutive equation through a mapping (homogenization) to the continuum-mechanical model. In return, the shortening velocity, ${\dot{\lambda }}_{f}\leq 0$, is passed from the continuum-mechanical model to the half-sarcomere model.

To take into account the length changes due to skeletal muscle tissue deformations, the bioelectrical field equations are solved on a deforming/moving domain. Thus, the equations describing the AP propagation along the muscle fibers have to be adjusted to the deformation. This can be achieved either by modifying the conductivity tensor or by solving the monodomain equation on the deformed geometry. In this contribution, the latter is employed.

In the following sections, the different parts of the computational framework, that is, the mechanical model (Section 2.1), the half-sarcomere model (Section 2.2), and the AP propagation model (Section 2.3), are introduced. Furthermore, implementation and high-performance computing aspects of the resulting multiphysics discretization schemes are presented in Section 2.4.

### 2.1. The Mechanical Problem

In continuum mechanics, the motion of a body *ℬ* is described by the placement function **χ** that assigns each point **X** in the reference configuration at time *t*_0_ a corresponding point **x** in the actual (deformed) configuration at time *t* > *t*_0_; that is, **x** = **χ**(**X**, *t*). The deformation of a body is commonly measured by the deformation gradient tensor

(1) $$\begin{array}{c} F∶=\frac{\partial \chi \left(X,t\right)}{\partial X}=\frac{\partial x}{\partial X} \end{array}$$

and the strain by the Green-Lagrangian strain tensor **E**∶ = (1/2)(**C** − **I**), where **C** = **F** ^*T*^ **F** is the right Cauchy-Green deformation tensor and **I** denotes the second-order identity tensor.

Inertia forces and body forces are assumed to be small compared to the forces acting in the muscle. Thus, the balance of linear momentum reduces to

(2) $$\begin{array}{c} \mathrm{div}T=0, \end{array}$$

where **T** denotes the Cauchy stress tensor. The Cauchy stress can be derived from the second Piola-Kirchhoff stress tensor, **S**, via a scaled covariant push forward operation: **T** = *J* ^−1^ **F** **S** **F** ^*T*^, with *J*∶ = det⁡**F** being the Jacobian.

The stress tensor (e.g., **T** or **S**) is derived from a constitutive equation. A constitutive equation characterizes the material behavior under load; that is, it relates the stress in a body to the strain. Skeletal muscle tissue is generally considered to be transversely isotropic and hyperelastic. Furthermore, muscle tissue is considered to be incompressible under physiological conditions. The second Piola-Kirchhoff stress tensor of a hyperelastic material can be derived from a strain energy function *W* defined per unit reference volume by

(3) $$\begin{array}{c} S=2\frac{\partial W}{\partial C}-pC^{-1}, \end{array}$$

with hydrostatic pressure *p* entering (3) as Lagrange multiplier associated with the incompressibility constraint *J* − 1 = 0; see, for example, [25].

For transversely isotropic materials, the strain energy function can be expressed in terms of the right Cauchy-Green deformation tensor and a second-order structural tensor *M* = **a**_0_ ⊗ **a**_0_, where **a**_0_ denotes a unit vector in the reference configuration pointing in the fiber direction. Applying the theory of invariants (see Spencer [26]), the strain energy function of a transversely isotropic material can be expressed as

(4) $$\begin{array}{c} W\left(C,M\right)=W\left(I_{1},I_{2},I_{3},I_{4},I_{5}\right), \end{array}$$

with principal invariants *I*_1_ = tr⁡**C**, *I*_2_ = (1/2)[(tr⁡**C**)^2^ − tr⁡(**C** ^2^)], and *I*_3_ = det⁡**C** = *J* ^2^ and mixed invariants *I*_4_ = tr⁡(**C** *M*) and *I*_5_ = tr⁡(**C** ^2^ *M*).

Following the idea of a fiber-reinforced material (cf. Spencer [27]), the strain energy function is split into an isotropic part *W* ^iso^ that represents the ground matrix and an anisotropic part *W* ^ani^ that represents the embedded fibers. Furthermore, a term *W* ^act^ is introduced to represent the muscle's ability to actively generate force via crossbridge cycling:

(5) $$\begin{array}{c} W\left(I_{1},I_{2},I_{3},I_{4},I_{5}\right)=W^{\text{iso}}\left(I_{1},I_{2}\right)+W^{\text{ani}}\left(I_{4},I_{5}\right)+W^{\text{act}}. \end{array}$$

On the right-hand side of (5), a dependence on the third principal invariant has directly been omitted due to the incompressibility constraint *I*_3_ = (det⁡**F**)^2^ = 1. Being based on the principle of superposition, the ansatz in (5) neglects any couplings between the individual parts of the strain energy leading to the assumptions that (i) the active behavior is independent of the other terms and (ii) there is no interaction between the fibers and the matrix.

In the following, first the terms representing the passive behavior of skeletal muscle are introduced before describing the active part of the strain energy.

#### 2.1.1. Passive Material Behavior

For the isotropic contribution, the Mooney-Rivlin material description is employed; see, for example, Holzapfel [28]. This material description is known to be well suited for representing *J*-like stress-stain curves of soft biological tissues:

(6) $$\begin{array}{c} W^{\text{iso}}\left(I_{1},I_{2}\right)=c_{10}\left(I_{1}-3\right)+c_{01}\left(I_{2}-3\right). \end{array}$$

The material parameters of the Mooney-Rivlin model, *c*_10_ and *c*_01_, are determined in a uniaxial compression test using the experimental data of Zheng et al. [29]. The set of parameters used within this work is summarized in Table 1. For the anisotropic contribution, a polynomial strain-energy function of the fiber stretch, $\lambda_{f}=\sqrt{I_{4}}>0$, has been adopted from Markert et al. [30]:

(7) $$\begin{array}{c} W^{\text{ani}}\left(\lambda_{f}\right)=\overset{N}{\underset{i=1}{\sum }}\left(\frac{b_{i}}{d_{i}}\left(\lambda_{f}^{d_{i}}-1\right)-b_{i}\ln\lambda_{f}\right), \end{array}$$

where *N* is the number of polynomial terms and *b*_*i*_ and *d*_*i*_ denote material parameters. Note that the anisotropic contribution applies only to the tensile range, that is, for *λ*_*f*_ > 1. A uniaxial extension test in fiber direction is used to fit material parameters *b*_*i*_ and *d*_*i*_ to the experimental data of Hawkins and Bey [31]. A single polynomial term (*N* = 1) was found to be sufficient to reproduce the experimental data. 

#### 2.1.2. Active Contractile Behavior

In many physiological conditions the mechanical behavior of skeletal muscle is dominated by its active, force generating behavior. In accordance with previously published skeletal muscle models [6, 15, 32], it is assumed that the active stress only acts in fiber direction. Furthermore, the generated force depends on the length of the muscle [33] and the shortening velocity [34]. Following this, the active part of the strain energy *W* ^act^ is assumed to be a function of deformation, represented through the fiber stretch *λ*_*f*_, the rate of deformation ${\dot{\lambda }}_{f}$, and the fiber direction. Proceeding from (3), the active part of the stress tensor yields

(8) Sact=2∂Wact∂C=2∂Wact∂λf∂λf∂I4∂I4∂C=1λf∂Wact∂λfa0⊗a0=Pactλfa0⊗a0.

In (8), the scalar-valued active stress function, *P* ^act^, which takes the form of a nominal (or engineering) stress, is introduced. Further, the active stress function, *P* ^act^, is assumed to depend on a constant maximum active stress *P* ^max⁡^ = 7.3 N/cm^2^ (cf. [31]), a function relating the generated stress to the muscle length *f*_*ℓ*_(*λ*_*f*_), and a function that links the macroscopic continuum-mechanical system to the quantities at the microscale $\bar{\gamma }$, which depends on the level of activation *α* and the velocity ${\dot{\lambda }}_{f}$:

(9) $$\begin{array}{c} P^{\text{act}}=P^{\max}f_{\ell }\left(\lambda_{f}\right)\bar{\gamma }\left(\alpha ,{\dot{\lambda }}_{f}\right). \end{array}$$

The function $\bar{\gamma }$ is determined in a biophysical model at the microscale (see Section 2.2). The force-length relation is adopted from Röhrle et al. [15] (see also [6]):

(10) fℓ(λf)={−254(λfλfopt)2+252λfλfopt−5.25,if  0.6≤λfλfopt≤1.40,otherwise.

In (10), *λ*_*f*_^opt^ denotes the optimal fiber stretch, which, based on experimental data [31], is assumed to take a value of 1.2.

In summary, the second Piola-Kirchhoff stress tensor, **S**, yields

(11) $$\begin{array}{c} S=S^{\text{iso}}+S^{\text{ani}}+S^{\text{act}}-pC^{-1},\\ S^{\text{iso}}=2c_{10}I+2c_{01}\left(I_{1}I-C\right),\\ S^{\text{ani}}=b_{1}\left(\lambda_{f}^{d_{1}-2}-\lambda_{f}^{-2}\right)a_{0}⊗a_{0},\\ S^{\text{act}}=\lambda_{f}^{-1}P^{\max}f_{\ell }\left(\lambda_{f}\right)\bar{\gamma }\left(\alpha ,{\dot{\lambda }}_{f}\right)a_{0}⊗a_{0}. \end{array}$$

### 2.2. The Micromodel of a Half-Sarcomere

The basis for modeling subcellular processes in the present contribution is the Shorten et al. [11] model. The Shorten model describes the complex, nonlinear, biophysical processes leading from electrical excitation to contraction and force generation of a half-sarcomere by means of ODEs. Two versions of the model using slightly different parametrizations allow the distinction between slow-twitch (type I) and fast-twitch (type II) muscle fibers. The model has been validated on mouse muscles.

To model the entire ECC, the half-sarcomere model [11] combines several submodels describing (a) membrane electrophysiology, (b) calcium release from the sarcoplasmic reticulum (SR), (c) calcium dynamics, (d) crossbridge dynamics, and (e) fatigue. In more detail, the individual parts are as follows. (a) For a description of the Hodgkin-Huxley electrophysiology of action potentials via ionic currents that pass through various channels and pumps (sodium channels, delayed rectifier and inverse rectifier potassium channels, chloride channels, and Na^+^-K^+^ pumps) in the sarcolemma and T-tubules, see Adrian and Peachey [35] and Wallinga et al. [36]. (b) Intracellular calcium release from the sarcoplasmic reticulum to the cytosol in response to membrane depolarization through RyR calcium release channels is described by a ten-state model originally proposed by Ríos et al. [37]. This submodel couples the T-tubule membrane voltage to the opening of the dihydropyridine receptor/RyR complex. (c) The released calcium (Ca^2+^) ions bind in the cytosol to parvalbumin and ATP along with troponin on the myofilaments. Moreover, intracellular magnesium ions (Mg^2+^) compete with Ca^2+^ for parvalbumin and ATP binding sites. After being transported back to the SR via Ca^2+^-ATPase, Ca^2+^ binds to calsequestrin. The description of the calcium dynamics goes back to the model of Baylor and Hollingworth [38]. (d) The binding of two Ca^2+^ ions to troponin C leads to a conformational change in the troponin molecule that removes the blocking tropomyosin from the actin filament and thereby allows the myosin head to attach to the actin binding sites. This model is based on an eight-state model of crossbridge dynamics in skeletal muscle using the generic models of Razumova et al. [39, 40] and Campbell et al. [41, 42]. (e) Muscle fatigue is modeled through subcellular mechanisms on the basis of phosphate dynamics. The accumulation of phosphate (P_*i*_) is believed to be the primary mechanism behind metabolic fatigue. Here, P_*i*_ is formed from the energy-providing reaction of ATP to adenosine diphosphate (ADP) during crossbridge cycling when weakly bound crossbridges isomerize into strongly bound crossbridges. The produced phosphate is transported passively to the SR where it precipitates with Ca^2+^ [11].

Although the degree of detail of the model of Shorten et al. [11], for example, modeling the signaling pathway of the ECC or fatigue, is not essential for the presented overall modeling framework, the authors refrain from simplifying the model, as this will be the basis for further developments that will build on different biophysical components. Moreover, the complexity of the model introduces new challenges for efficiency and parallelization.

According to the sliding filament theory [43], the active force production in skeletal muscle is due to crossbridge cycling. The crossbridge dynamics model, which depends on all above-described models, defines the force producing step called power stroke as the transition between the two attached states, that is, the prepower stroke state *A*_1_ and the postpower stroke state *A*_2_. Therefore, one can assume that the actively generated stress in a half-sarcomere under isometric conditions is proportional to the concentration of crossbridges in the postpower stroke state *A*_2_ [40]. The value of *A*_2_ is normalized using the value of *A*_2_ at maximum tetanic stimulation *α* ^max⁡^; that is, *A*_2_(*α*)/*A*_2_(*α* ^max⁡^)∈[0,1].

The half-sarcomere model [11] was developed for isometric contractions. Truly isometric conditions, however, do not exist in skeletal muscle, since (i) contractile tissue is in series with elastic components of the musculoskeletal system stretching under contraction-induced stress increase and (ii) various nonuniformities exist along the muscle fiber; that is, while one part of the fiber shortens, another part is stretched.

The scaling quantity *γ*, (cf. (9)) is found by multiplying the normalized concentration of crossbridges in the postpower stroke state by Hill's hyperbolic force-velocity relation [34]:

(12) $$\begin{array}{c} \gamma =\frac{A_{2}\left(\alpha \right)}{A_{2}\left(\alpha^{\max}\right)}\left[\frac{F^{\text{iso}}+a}{b-{\dot{\lambda }}_{f}}b-a\right]. \end{array}$$

In (12), *F* ^iso^ denotes the maximum isometric active force, and *a* and *b* are the Hill parameters, which are chosen such that *a*/*F* ^iso^ = 0.25 [44, 45] and $b/{\dot{\lambda }}_{f}^{\max}=0.25$ [46] with ${\dot{\lambda }}_{f}^{\max}$ being the maximum shortening velocity at zero force production.

To extend the single half-sarcomere model to a model of a muscle fiber, the electrophysiological characteristic of propagating APs along the length of fibers is considered. The equations representing the AP propagation are presented in Section 2.3.

### 2.3. Action Potential Propagation

The propagation of an AP along a skeletal muscle fiber is initiated at the neuromuscular junction located in the middle of the length of each fiber. Starting at the neuromuscular junction, the short-term depolarization of the muscle-fiber membrane voltage travels along the length of the fiber towards its ends.

The macroscopic electrical conductivity of muscle tissue perpendicular to the fiber direction is up to one magnitude lower than the conductivity along the fiber direction [47, 48], and electrical stimulation from one fiber to adjacent ones is not observed. Therefore, the propagation of an AP along a skeletal muscle fiber is modeled as a 1D system. The propagation of APs in biological tissue is typically modeled using the bidomain equations; see, for example, Pullan et al. [49]. In the 1D case, the bidomain equations reduce to the simpler monodomain equation, a reaction-diffusion equation [50, 51], which is given by

(13) $$\begin{array}{c} \frac{\partial }{\partial s}\left(\sigma \frac{\partial V_{m}}{\partial s}\right)=A_{m}\left(C_{m}\frac{\partial V_{m}}{\partial t}+I_{\text{ion}}\right). \end{array}$$

In (13), *s* denotes the spatial variable describing the position along the path of the fiber, *σ* is the conductivity, *V*_*m*_ represents the membrane voltage, *A*_*m*_ reflects the ratio of the membrane surface area to the volume, and *C*_*m*_ is the capacitance of the cell membrane per unit area. Depending on the twitch type of the fiber, two different values are used for the membrane capacitance, that is, *C*_*m*_ = 0.58 *μ*F/cm^2^ for slow-twitch fibers and *C*_*m*_ = 1.0 *μ*F/cm^2^ for fast-twitch fibers [11]. The value of *A*_*m*_ = 500 cm^−1^ is identical for both fiber types [52]. Furthermore, the reaction term *I*_ion_ depends nonlinearly on *V*_*m*_ and denotes the sum of ionic currents crossing the cell membrane of the sarcolemma and the T-tubule.

### 2.4. High-Performance Computing

After introducing the individual submodels and their interactions, this section focuses on efficient solution strategies for this complex and computationally very demanding multiphysics model describing phenomena on different length and time scales. To achieve this, various concepts of software engineering, for example, advanced discretization schemes for multiphysics problems, parallelization, or staggered solution schemes, are adopted. These concepts have been implemented within the open-source software library OpenCMISS [19].

#### 2.4.1. Operator Splitting

For the numerical treatment of the monodomain equation (cf. (13)), it is convenient to apply an operator splitting technique (or fractional-step method) to separate the nonlinear reaction term from the diffusion term; see, for example, Sundnes et al. [50, 53]. Applying the first-order accurate Godunov-type splitting, (13) yields

(14a) $$\begin{array}{c} \frac{V_{m}^{*}-V_{m}^{k}}{\Delta t}=-\frac{1}{C_{m}}I_{\text{ion}}\left(V_{m}^{k}\right), \end{array}$$

(14b) $$\begin{array}{c} \frac{V_{m}^{k+1}-V_{m}^{*}}{\Delta t}=\frac{1}{A_{m}C_{m}}\frac{\partial }{\partial s}\left(\sigma \frac{\partial V_{m}^{k+1}}{\partial s}\right), \end{array}$$

where Δ*t* refers to the time step, *V*_*m*_^*k*^ and *V*_*m*_^*k*+1^ denote the values of the membrane voltage at discrete times *k*Δ*t* and (*k* + 1)Δ*t*, respectively, and *V*_m_* is the value at the intermediate time t*. The advantage of the operator-splitting approach is that different numerical methods can be applied to the different subsystems; that is, the nonlinear reaction (14a) is solved using an implicit multistep ODE integration method as commonly done for highly nonlinear, stiff, biophysical cell models (see Pullan et al. [49]), while one uses the backward-Euler method for the diffusion equation (14b). Furthermore, different time steps can be used for the different subsystems (subcycling). For the discretization of the spatial derivative term in (14b) the finite element method (FEM) [54] is applied.

#### 2.4.2. Discretization in Space and Time

The solution of the bioelectrical field equations, (14a) and (14b), requires an extremely small time step and a very fine mesh due to the rapid changes and steep gradients occurring in physiological cell models; see [49, 52]. On the other hand, using a similarly spatial and temporal discretization for the solution of the 3D mechanical model is prohibitively expensive and unnecessary, as changes on the scale of an entire muscle occur at considerably larger time scales.

Following the idea of different characteristic length scales, a multiphysics discretization scheme is proposed: a much finer mesh is used for the bioelectrical model than for the continuum-mechanical system. First, a relatively coarse 3D finite element (FE) mesh of the muscle's geometry is generated. Then, relatively fine 1D FE muscle fiber meshes are embedded in the 3D elements (cf. [18]). The governing equations of the continuum-mechanical model, (2), and the incompressibility constraint are discretized using the coarse 3D mesh, while the diffusion part of the bioelectrical field equation, (14b), is solved on the 1D fiber meshes. Some variables exist on both meshes, and thus, transfer operations between the two meshes are required. The transfer from the coarse 3D FE mesh to the fine 1D fiber meshes is called interpolation, while the transfer in the opposite direction is termed homogenization. The homogenization and interpolation processes are discussed for each affected variable in Section 2.4.3.

**Figure 2:**
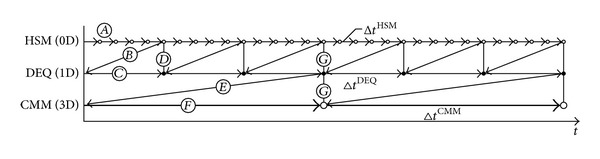
Time-stepping scheme, where Δ*t* is the time step, HSM denotes the half-sarcomere model, DEQ is the diffusion equation, and CMM is short for the continuum-mechanical model.

Due to the different characteristic time scales of the different physical phenomena, a staggered solution scheme with three different time steps is applied in this work. A schematic representation of the time-stepping scheme is shown in Figure 2. First, the half-sarcomere models, (14a), are solved for 50 time steps with time step size Δ*t* ^HSM^. The symbol (*A*) in Figure 2 denotes the solution process for computing the states of the half-sarcomere model for time *t* + Δ*t* ^HSM^. Note, for simplicity and readability of Figure 2, only a fractional number of time steps are depicted. In case of computing the cellular states, which will be used within the next time step of the diffusion equation, only 5 instead of the actual 50 time steps are depicted in Figure 2. Each discretization point of the monodomain equation is associated with its own half-sarcomere model. The half-sarcomere model is mathematically described by ODEs in time, which do not rely on any spatial quantities. Therefore, each half-sarcomere model can be solved independently of all other half-sarcomere models. The final values of the membrane voltage computed in these steps are used as starting values for the diffusion equation (14b). This process is denoted by (*B*) in Figure 2. Following the solution of the diffusion equation (14b) with time step Δ*t* ^DEQ^, which is indicated by (*C*) in Figure 2, the updated values of the membrane voltage are used as initial conditions for the next solution step of the half-sarcomere model (indicated by (*D*) in Figure 2). This procedure is repeated a number of times (3 times in Figure 2, 1000 times in the actual computations) before the values of the active stress *γ* are homogenized ($\gamma \to \bar{\gamma }$). The homogenization process is denoted in Figure 2 by (*E*). The homogenized values $\bar{\gamma }$ enter the continuum-mechanical model, (2), through the stress tensor, which is given by (11). The continuum-mechanical model is only solved in time increments of size Δ*t* ^CMM^ (cf. step (*F*) in Figure 2). Further, the values of the sarcomere velocity are interpolated and applied to the half-sarcomere models; see (*G*). At the same time, the position of the nodes of the 1D fiber meshes is updated based on the calculated deformation. The described steps are repeated until the final time is reached.

#### 2.4.3. Homogenization and Interpolation

As described above, some variables are shared between the different discretizations. For example, the values of the active stress field are determined in the model of the half-sarcomere, that is, at the nodes of the 1D fiber meshes. In order to include the active stress field in the continuum-mechanical constitutive equation, which is evaluated at the integration points, for example, the Gauß points, associated with the weak formulation of the 3D finite elements, the values need to be homogenized. Like in Röhrle et al. [15], the homogenization is achieved by computing the arithmetic mean of all 1D nodal values that are closest to a certain Gauß point of the continuum-mechanical 3D FE mesh. Other elaborate homogenization techniques like those proposed in [55, 56] could be adopted but are not further considered here.

The positions of the nodes of the 1D fiber meshes are defined in terms of the local element coordinate system of the 3D geometric FEs. Using this definition, their actual positions can be determined from the deformation of the muscle's geometry, that is, from the actual configuration. Using the basis functions of the 3D FEs for the interpolation, the nodal positions of the 1D fiber meshes are updated after each solution of the mechanical submodel.

Further, information about sarcomere velocity is required in the half-sarcomere models located at the nodes of the 1D fiber meshes; see (12). The sarcomere velocity cannot be determined in the biophysical model of the half-sarcomere, as the velocity also relies on the boundary conditions of the continuum-mechanical model of the entire muscle. Therefore, the local sarcomere velocity ${\dot{\lambda }}_{f}$ is approximated by a backward finite difference scheme: ${\dot{\lambda }}_{f}=(\eta_{i}^{k+1}-\eta_{i}^{k})/\Delta t^{\text{CMM}}$, where *η*_*i*_ represents the distance between two adjacent nodes and *k* and *k* + 1 denote two consecutive time steps of the continuum-mechanical model. To avoid unrealistic high variations in sarcomere velocity and to mimic the structural links between adjacent skeletal muscle fibers, the average of the velocity is calculated over a patch of seven sequential nodes of one fiber.

#### 2.4.4. Data Structure

**Figure 3:**
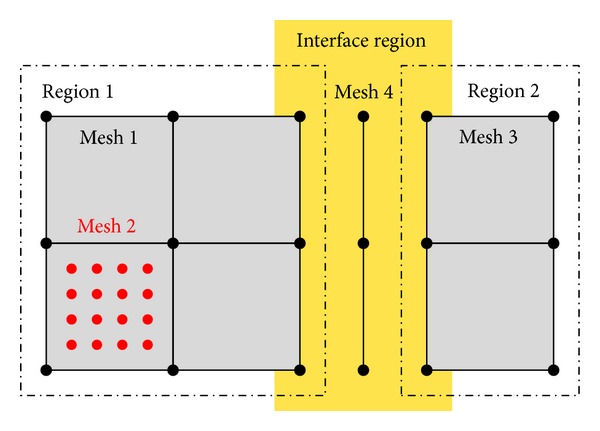
Schematic drawing of regions and meshes in OpenCMISS. Different regions can be coupled via interface conditions. Several meshes can be associated with a region.

The open-source software library OpenCMISS [19] provides a highly flexible framework for the simulation of coupled multiphysics problems. Being arranged in a hierarchical fashion, the concepts of regions, meshes, fields, and so forth (see [19] for details) allow for couplings between different physical problems at different length and time scales. The presented skeletal muscle model is built on a single region, since the different physical models occupy the same space (volume-coupled problem). When the interaction of a skeletal muscle with neighboring structures such as other muscles, bone, fat, or skin is of interest, these structures can be added to the model as additional regions; see Figure 3. To couple different regions, their interaction can be defined via interface conditions, for example, contact. 

The region used for the chemoelectromechanical muscle model contains two meshes: a 3D representation of the geometry that is used for the continuum-mechanical model (mesh 1 in Figure 3) and a second mesh (mesh 2 in Figure 3) consisting of a number of 1D fibers that are used for the solution of the bioelectrical model. The 1D fiber meshes are embedded in the 3D FEs.

Fields are a key data structure in OpenCMISS. Any quantity that can be associated with a mesh is represented in OpenCMISS as a field. A field variable can be constant across the mesh, it can vary from element to element, from node to node, from interpolation point (e.g., Gauß point) to interpolation point, or from data point (arbitrarily located) to data point. The representation of fields in OpenCMISS is based on FieldML [20], which provides field transfer operators (homogenization or interpolation) to handle different spatial scales; see also Section 2.4.3.

Further, OpenCMISS employs nested control loops to handle different temporal scales. In the presented model, two separate control loops for the continuum-mechanical model and the bioelectrical problem, each with its own time step size, are linked to a superior main control loop. The control loop for the mechanical model is only associated with a single solver, while the bioelectrical control loop is connected to a solver for the diffusion equation and a second solver for the half-sarcomere model.

The half-sarcomere model is provided in CellML format [21]. CellML is a markup language for the description of subcellular models based on XML (Extensible Markup Language). In a multiscale model, CellML can be used to conveniently describe the physical processes occurring at a single point within a model at a larger spatial scale. A CellML model repository containing more than 500 models is available for download at http://www.cellml.org/, among them the biophysical model of a half-sarcomere of Shorten et al. [11]. In OpenCMISS, the time step sizes for the CellML models can be chosen independently of the time step sizes used to solve equations representing different physics. For example, the half-sarcomere model, (14a), requires a much smaller time step than the diffusion equation, (14b), and hence, subcycling of the CellML model is employed.

#### 2.4.5. Parallelization

OpenCMISS is developed for parallel computations in a heterogeneous multiprocessing environment [19], where the MPI standard (http://mpi-forum.org/) is used for distributed memory parallelization and the OpenMP standard (http://openmp.org/) is used for shared memory parallelization.

**Figure 4:**
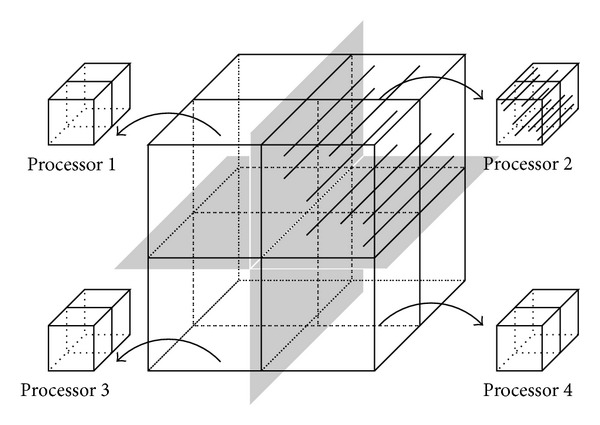
Schematic drawing of the domain decomposition as realized for the chemoelectromechanical skeletal muscle model. The decomposition of the 3D mesh of the muscle geometry does not split any of the muscle fiber meshes.

The implementation of the distributed memory parallelization in OpenCMISS builds on the concept of domain decomposition. For the presented chemoelectromechanical skeletal muscle model, the domain is decomposed in such a way that each 1D embedded fiber mesh is uniquely assigned to a processor; see Figure 4. This approach reduces the amount of communication between the individual processors to a minimum for the bioelectrical model. Parallel efficiency is hereby guaranteed by the fact that the diffusion part of the bioelectrical model is evaluated 1000 times more often than the continuum-mechanical model (Δ*t* ^CMM^ = 1000  Δ*t* ^DEQ^). Hence, a user-defined domain decomposition, rather than a computed decomposition based on the graph partitioning packages ParMETIS (http://glaros.dtc.umn.edu/gkhome/metis/parmetis/overview) or Scotch (http://www.labri.fr/perso/pelegrin/scotch/), which is typically used within OpenCMISS, is optimal with respect to the entire chemoelectromechanical model.

Although currently not implemented, the individual muscle fiber meshes within a single computational domain could be further parallelized using an OpenMP shared memory parallelization. Further, the integration of the ODEs describing the half-sarcomere model is highly suitable for parallel execution on GPGPUs.

## 3. Results

### 3.1. Computational Model

To analyze the performance of the computational framework, a simple geometric model is considered. A cubic geometry with 2 cm edge lengths is generated and discretized using eight triquadratic/trilinear Lagrange finite elements (Taylor-Hood elements). A fiber direction is defined that is uniformly aligned and parallel to an edge of the cube. A total of 400 muscle fiber meshes are evenly distributed in the cubic geometry, and each fiber is discretized using 60 linear Lagrange finite elements.

First, the muscle is passively stretched in fiber direction by 20% to reach the optimal fiber stretch of *λ*_*f*_^opt^ = 1.2. Under isometric conditions (the muscle specimen is fixed at the optimal length), a 100 Hz tetanic stimulation frequency is applied to the central half-sarcomere model of all fibers in the model.

To analyze the speedup in a parallel environment, the described model is executed on 1, 2, and 4 processors. A speedup of 2.18 is achieved when going from 1 to 2 processors, while a speedup of 1.95 is achieved when comparing 2 to 4 processors. Further, the simulations were repeated using only 36 1D fiber meshes instead of 400. In this case, a speedup of 1.44 is achieved when going from 1 to 2 processors, while a speedup of 1.50 is achieved when comparing 2 to 4 processors.  Table 2 lists the timing results and speedup factors for an Intel Xeon Processor E5520 and 8 GB of RAM.

In the example with 400 fibers, the solution of the bioelectrical model dominates the total computing time. Here, a speedup factor of 2.18, which exceeds the theoretically achievable value of 2, occurs, which can be explained by a significantly higher number of cache misses on 1 processor than on multiple processors, as the size of the bioelectrical model for each processor scales down proportionally to the number of processors. (No ghost elements exist, and no communication between the processors is required in the bioelectrical model.) The other end of the spectrum is marked by the example using only 36 fibers, that is, 3 × 3 fibers per 3D element, leading to a one by one correspondence between the number of Gauß points in the plane perpendicular to the fibers and the number of embedded fibers. (The 3D elements use 3 × 3 × 3 Gauß points.)

Note that the discretization for the mechanics is independent of the number of embedded fibers and is identical in both cases. In case of 36 fibers, the speedup factors are very poor, since the solution of the continuum-mechanical problem claims a larger fraction of the total computing time. The poor scaling of the continuum-mechanical model is due to the few 3D elements. Together with the required ghost elements each processor has to compute (i) 8 FEs when 1 processor is used, (ii) 8 FEs when 2 processors are used, and (iii) 6 FEs when 4 processors are used. (All elements that share a surface with an actual element of the domain are ghost elements.) For practical applications, however, a finer discretization of the continuum-mechanical model is desirable to achieve a higher accuracy and a better approximation of the muscle's geometry. Furthermore, the application of more fibers is preferable for a realistic muscle simulation.

**Figure 5:**
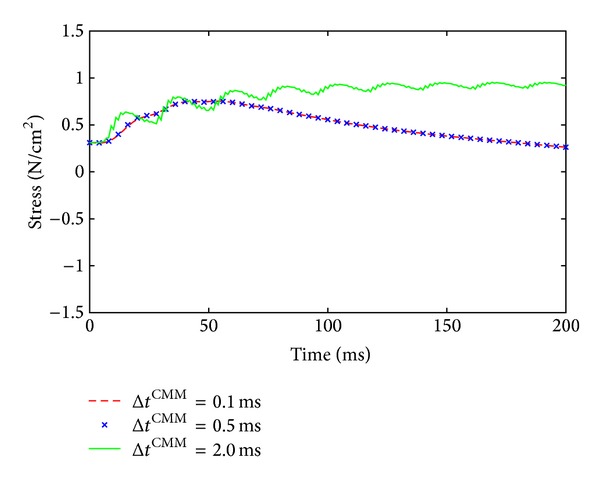
Model behavior for different time step sizes of the continuum-mechanical model, Δ*t* ^CMM^. The solutions for the smaller two time steps almost coincide (red dashed line and blue crosses), while the solution for the largest time step size shows a nonphysical, oscillatory behavior.

Within this work, different time step sizes are used for the solution of the different submodels. Critical time step sizes for the bioelectrical model have already been investigated in Davidson [52]. Here, the model behavior for different time step sizes of the continuum-mechanical model, Δ*t* ^CMM^, are investigated.  Figure 5 shows the stress evolution of a shortening contraction (*v* = 0.1*v*_max⁡_) of a muscle that is uniformly stimulated at 50 Hz. The results for three different time step sizes (Δ*t* ^CMM^ = 0.1 ms, 0.5 ms, and 2.0 ms) are shown, whereof the solutions for the smaller two time steps almost coincide (red dashed line and blue crosses) and the solution for the largest time step size (Δ*t* ^CMM^ = 2.0 ms) depicts significant deviations and oscillatory behavior.

### 3.2. Force-Velocity Relation

**Figure 6:**
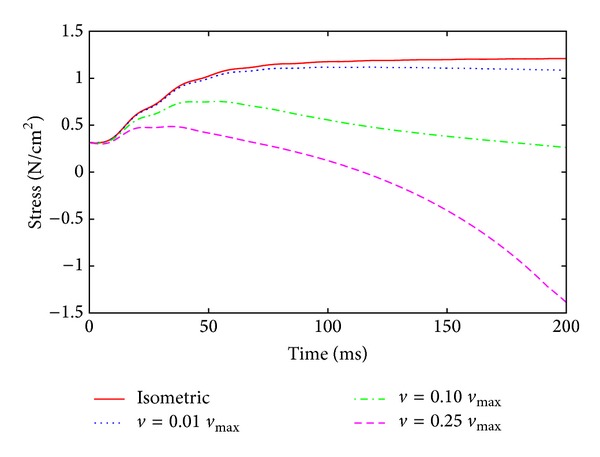
Force-velocity behavior I. Shown are shortening contractions at four different velocities using the fully coupled chemoelectromechanical skeletal muscle model.

Under nonisometric conditions, the force-velocity relation plays an important role in skeletal muscle simulations. To illustrate the influence of the velocity on the force, a geometrically simple model is examined. Again, a rectangular tissue block with uniform fiber direction and 2 cm length is first stretched in fiber direction by 20% to reach the optimal muscle length. In a second step, all fibers are jointly stimulated with 50 Hz, and the muscle specimen is allowed to shorten at a certain velocity *v*. The numerical experiment is repeated under isometric conditions and at 1, 10, and 25% of the maximum shortening velocity of 200 mm/s. The results are depicted in Figure 6. The model predicts lower forces at higher velocities.

**Figure 7:**
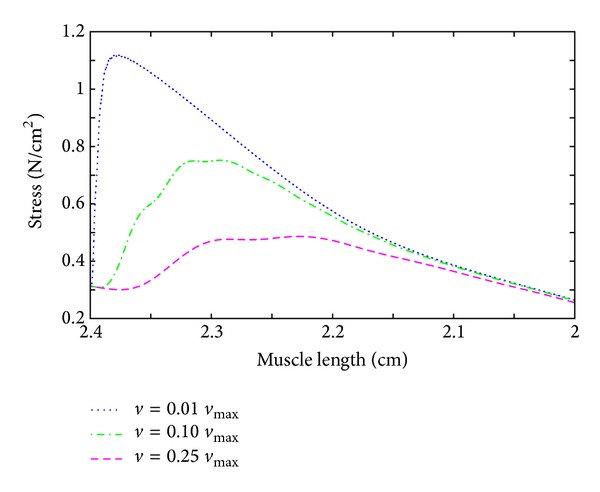
Force-velocity behavior II. For three different shortening contractions, the stress is plotted versus the actual muscle length.

The decline in the force by choosing a shortening velocity of 10 and 25% of the maximum shortening velocity is a direct result of the force-length relationship and due to the fact that the muscle reaches for higher velocities lengths at which it can produce much less force in a shorter amount of time. To segregate the influence of the force-length relation, Figure 7 shows the same results as Figure 6, however by plotting the force versus the actual length of the specimen.

### 3.3. Feasibility of the Framework and Code

**Figure 8:**
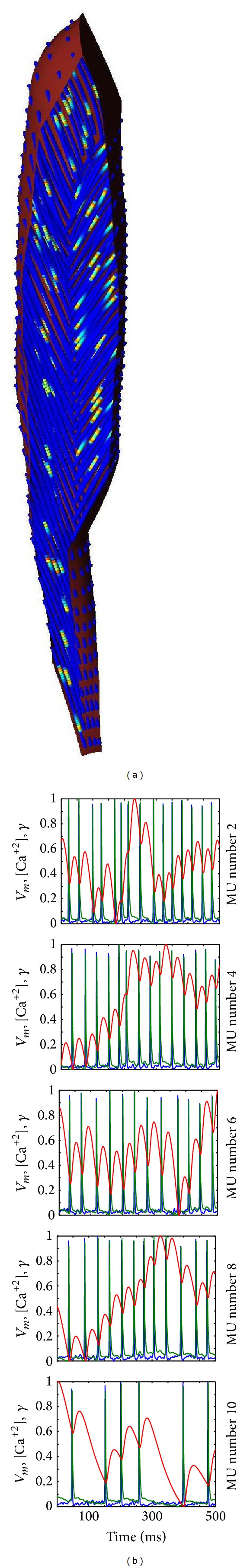
Tibialis anterior muscle. Shown is the geometry of the muscle and the fiber distribution, where the fibers indicate the local membrane potential in color (a). The normalized muscle fiber membrane voltage *V*_*m*_ (blue), normalized free calcium concentration in the myoplasm [Ca^2+^] (green), and normalized active stress *γ* (red) are plotted versus time for motor units 2, 4, 6, 8, and 10 (b).

To demonstrate the ability of the chemoelectromechanical model to represent a realistic muscle, a model of a tibialis anterior (TA) muscle is generated. The geometrical representation of the TA is based on the Visible Human data set [57], and the fiber direction is based on diffusion tensor MRI data. The geometric model has previously been used in Röhrle et al. [18]. Within the present contribution, 10 MUs and stimulation frequencies between 6 and 30 Hz are assumed for the TA model. Detailed information on the methodology of assigning MU fiber distributions is given in [18]. The motor endplates are assumed to be located at the center of the fibers, where a depolarizing current is injected at the times of stimulation. The numerical experiment is carried out under isometric conditions.  Figure 8 shows the geometry of the TA muscle and the fiber distribution (a). The fibers show the local membrane potential distribution (blue indicates the resting potential, red indicates the depolarized state). Further, the normalized muscle fiber membrane voltage (blue), normalized free calcium concentration in the myoplasm (green), and the normalized active stress (red) are plotted versus time for MUs 2, 4, 6, 8, and 10 (see Figure 8(b)).

## 4. Discussion

From a modeling point of view, this work appeals to a very complex biophysical half-sarcomere model describing the entire EEC. The model contains a large number of parameters. Many of these parameters are difficult to determine, and only few are available for any muscle and any species. The most trustworthy parameter sets are probably given by Shorten et al. [11], who validated their model to experimental data for different electrical stimulation patterns on force production in soleus and extensor digitorum longus (EDL) muscles of mice for slow-twitch and fast-twitch fibers, respectively. Using in the proposed multiscale framework the described detailed biophysical model provides the basis for testing different physiological hypotheses and investigating different skeletal muscle phenomena, such as fatigue, signaling pathways, residual force enhancement/depression, myopathies, or influence of drugs in future studies.

Although the ECC model of Shorten et al. [11] describes many aspects of the entire pathway from electrical stimulation to force production, it does not consider the titin filament that has recently gained attention in the literature [58, 59]. Nevertheless, a model representing the effect of the titin filament, for example, the one by Rode et al. [60], could be included in the model of Shorten et al. [11] if conditions are of interest, where the titin filament is expected to have a significant influence.

Further, the modeling assumption that a fiber can be represented as a 1D geometrical object assumes that all parallel aligned sarcomeres within the cross section of a fiber behave identically not allowing for sarcomere inhomogeneities within the cross section of the fiber. Moreover, the embedding of the anatomically based 1D fiber meshes within the 3D mesh for the continuum mechanics and the homogenization process required due to the different meshes provide a few restrictions on the micromechanical skeletal muscle model. While assuming the electrical isolation of individual fibers is physiologically valid, the proposed framework does not distinguish individual fibers or fascicles in the mechanical model. While there exist first works on investigating the mechanical interaction of adjacent muscle fibers and fascicles through the extracellular connective tissue, for example, by Sharafi and Blemker [61, 62], the mechanical behavior of the fibers and the connective tissue within this framework is based on a macroscopic continuum-mechanical approach. Including micromechanical considerations within this framework, however, would lead to a computationally extremely demanding muscle model. This is particularly due to the fact that the mechanical considerations of Sharafi and Blemker [61, 62], which have only been carried out on a small block of tissue, are carried out for purely passive muscle tissue and would need to be further extended to active contractile behavior. Furthermore, material parameters of the extracellular connective tissue and stripped muscle fibers are not readily available [61], and hence a further source of uncertainty would be introduced into the model.

Within this framework, the active stresses determined in the half-sarcomere model are homogenized and included in the continuum-mechanical constitutive equation. The homogenization is required for computational efficiency. A skeletal muscle model that would use the same number of elements for the bioelectrical and the mechanical problem no longer require any homogenization; however, this approach results in a computational model that is no longer feasible for any practical application. It should be noted that the homogenization process has little effect on the convergence behavior of the mechanical problem. This has been demonstrated in Röhrle et al. [15] by maintaining a fixed number of embedded fiber models while successively refining the number of 3D mechanical elements until homogenization is no longer required. The investigation showed very good convergence properties [15] if compared to the mechanical-only problem.

Improving the constitutive equation for describing the macroscopic behavior of skeletal muscle mechanics does not only apply to its active contribution. In general, future research needs to further focus on experimental studies and continuum-mechanical material descriptions to develop valid constitutive equations for skeletal muscle mechanics in general. Within this framework the isotropic Mooney-Rivlin material model has been extended by a contribution acting in the along-fiber stretch regime for describing the transversely isotropic material behavior of passive muscle tissue. The anisotropic contribution to the passive behavior is negligibly small in the small strain regime, and hence the Mooney-Rivlin parameters can be used to characterize the passive material behavior around the reference configuration. Based on a comparison with the infinitesimal strain theory, the consistency condition for the Mooney-Rivlin parameters yields a value of *μ* = 2(*c*_10_ + *c*_01_) ≈ 7 kPa for the shear modulus [28], which is close to experimentally determined values [63]. However, there is some experimental evidence that under compression passive muscle tissue exhibits a stiffer behavior in the cross-fiber direction than in the fiber direction [64]. Although this material behavior can be included in a continuum-mechanical formulation [64], the material behavior of the present contribution is isotropic in the compressive range and exhibits a transversely isotropic material behavior in the along-fiber stretch region, as in most other works in this field of research; see, for example, [6, 15]. More accurate or micromechanically based subject- or muscle-specific material parameters would be desirable but are currently not available.

Despite using two different discretizations, that is, one grid for the mechanical model and a different grid for the electrophysiological model, the computational cost of the model is still very considerable. Hence, a staggered solution is proposed to further reduce the computational effort. Staggered solution schemes are often favorable when within one model different subsystems describe processes with very different characteristic time step sizes. The microscopic half-sarcomere model shows rapid changes and steep gradients, while the changes in the continuum-mechanical system occur at a much larger time scale. The application of the staggered solution scheme implies the following assumptions. The changes in the variables in the bioelectrical field equations, (14a) and (14b), are small within one time step of the continuum-mechanical model; that is, these changes do not have a strong effect on the continuum-mechanical system. On the other hand, the changes introduced through one solution step of the mechanical system, (2), are small; that is, not updating the mechanical fields at every time step at which the bioelectrical field equations are solved introduces a rather small error (cf.  Figure 5). Based on the results depicted in Figure 5, the time step for the considered problem could be chosen even larger; however, the authors have retained from this possibility as they have an extended framework in mind that also provides feedback from the mechanical to the recruitment model. In that case, it is presumed that a smaller mechanical time step might be more suitable. This, however, has to be shown in future research. Equivalent assumptions have to be made for the operator split within the bioelectrical field problem, where the diffusion equation is separated from the reaction term.

In contrast to staggered schemes, monolithic solution schemes do not rely on these assumptions. A monolithic scheme has been investigated for the bioelectrical field equations [65]; however, only a simple, phenomenological model for the reaction term has been evaluated. Further, Göktepe and Kuhl [66] propose a fully implicit approach for cardiac electromechanics. As the proposed chemoelectromechanical model uses a much more detailed, biophysical half-sarcomere model for the reaction term, the staggered schemes have been employed to reduce the overall cost while maintaining accuracy and stability for long stimulation periods. Further, the fact that the bioelectrical model is solved on a deforming domain (as a result of the continuum-mechanical model) results in monolithic solution schemes that are not so straightforward to implement.

## 5. Conclusions

An extensible, flexible, multiscale, and multiphysics modeling framework for nonisometric skeletal muscle mechanics has been presented. The skeletal muscle model spans the entire excitation-contraction pathway using an electrophysiological membrane model, a biophysical half-sarcomere model (including the hyperbolic force-velocity relationship) for active force generation, action potential propagation along individual muscle fibers, and a continuum-mechanical description of the macroscopic muscle tissue allowing for complex interactions with surrounding tissues. The framework is based on state-of-the-art parallelization techniques providing the basis to investigate many different aspects of skeletal muscle physiology and mechanics in the future. In particular, the extensible and flexible open-source software library OpenCMISS will provide the basis for future extensions such as including the effects of titin, neurocontrol, feedback mechanisms, and many more aspects. The key to all of that is its implementation within a single framework using novel data structures, for example, FieldML and CellML, not requiring any external data exchange, staggered solution schemes addressing computational efficiency in the presence of different and separable time scales, and parallelization strategies.

## Figures and Tables

**Table 1:** Parameters of the passive part of the constitutive equation.

*c*_10_	*c*_01_	*b*_1_	*d*_1_
6.352e^−10^ kPa	3.627 kPa	2.756e^−5^ kPa	43.373 [—]

**Table 2:** Execution time in seconds and resulting speedup for 1, 2, and 4 processors.

No. of processors	36 fibers		400 fibers	
	Time [s]	Speedup [—]	Time [s]	Speedup [—]
1	10004.32		177759.11	
2	6940.91	1.441	81360.24	2.185
4	4625.88	1.500	41763.99	1.948
